# Epilepsy and Other Convulsive Affections; Their Pathology and Treatment

**Published:** 1859-01

**Authors:** 


ART. III.—*Epilepsy and other Convulsive Affections, their Pathology and Treatment.* By CHARLES BLAND RADCLIFFE, M.D., Physician to the Westminster Hospital, etc. Second edition. 8vo., pp. 383. Churchill: London, 1858.

THE writer of this work, a bold and original thinker, evidently belongs to a class which, happily for the interests of practical medicine, is daily extending its ranks. He undertakes the investigation of certain important diseases of the organs of animal life, with a constant and careful reference to the physiology as well as the pathology of these organs. Convinced, as he assures us, that a sound physiology must ever go before a sound pathology, he precedes his remarks upon epilepsy and other convulsive disorders by certain considerations respecting the physiology of muscular motion. In fact, he divides his work into two parts essentially distinct, but mutually illustrative and explanatory. The first part occupies one-third of the volume, and is a thoughtful and elaborate attempt to demonstrate and establish a new theory of muscular contraction; a theory quite opposed to the prevailing one upon this subject, which, according to Dr. Radcliffe, involves a radical error, whose correction is absolutely necessary before we can attempt the satisfactory interpretation of epileptic disorders. The second part of the work is an essay of 254 pages upon the pathology, periodicity, and treatment of epilepsy and allied diseases. These two parts, however, are so arranged that it is optional with every one who may so choose, to read the practical portion of the work first, and then to turn back and ascertain how far the conclusions to which he is conducted by arguments of a purely pathological character—arguments which are complete in themselves—are borne out by the physiological portion.

The physiology being put forth as a novel basis for the pathology and therapeutics of the disease mentioned, we propose to present our readers with as complete a summary of it as our limits will admit.

The opinion at present entertained by most physiologists concerning muscular contraction is, that muscles are endowed with a vital property of contractility, and that muscular contraction is brought about when this property is called into action by certain agents, such as electricity or nervous influence. It is supposed, indeed, that this vital property of contractility is roused or excited or stimulated into a state of action when the muscle contracts, and hence the agents which thus rouse or excite or stimulate, are called stimuli. Haller, Hunter, Weber, Bowman, Burnett and others, have all, by their varied observations and experiments, endeavored to establish the physiological doctrine, now generally received, that this contractility is a vital property inherent in the living muscular fibre, and without which the latter would not be muscle. "This general property of contractility," writes Dr. Carpenter, "shows itself under two forms, which are alike distinct in the mode of their action, and in the conditions requisite for its excitation. Its most obvious and striking manifestations present themselves in the voluntary muscles and in the heart, which, when in activity, exhibit powerful contractions, tending to alternate with relaxations. The modification of contractility which is concerned in producing these, is distinguished as irritability. On the other hand, we find that the muscles exhibit a tendency to a moderate and permanent contraction, which is not shown by them when they are dead, and which cannot, therefore, be the result of elasticity, or of any simple physical property, and this contraction, instead of being consequent upon stimulation through the nerves, is especially excited by changes of temperature in the tissue itself. This endowment, which seems to exist in the greatest amount in certain forms of the non-striated muscle, is called tonicity." According to Bowman, "the contraction resulting from the exercise of the contractile force will be permanent, if no force from without be exerted to obliterate it by stretching; for a contracted muscle has no power of extending itself; there is no repellent force between its molecules." In opposition to these views, Dr. Radcliffe endeavors to show "that muscle contracts, not because it is stimulated to contract by nervous influence or electricity, or any other so-called stimulus of contraction, but because something has been withdrawn from the muscle which previously prevented the free action of common molecular attraction." This revolutionary doctrine he announced for the first time in 1851, in a work entitled "The Philosophy of Vital Motion." Four years later he returned to this interesting subject, in the first edition of the volume now under consideration, and giving more prominence to the pathological bearing of the theory, endeavored to show that epilepsy and all other disorders in which muscular contraction is in excess, are only intelligible when they are interpreted by such a theory of muscular motion.

In thus thinking that a great change is demanded in the theory of muscular action—a change which evidently amounts to no less than a complete revolution—our author does not stand alone; for it appears that Professors Matteucci of Pisa, Engel of Zurich, and Stannius of Rostock, have been led, by different circumstances, to entertain very similar ideas—at least so far as concerns the action of nervous influence in muscular motion.

According to Matteucci the nervous fluid "keeps the elements of muscular fibre in a state of repulsion analogous to that exhibited by electrified bodies. When this nervous fluid is no longer in a free state in the muscle, the elements of the latter are drawn together, as happens in cadaveric rigidity. The contraction is more or less decided, according to the quantity of fluid which disappears from the muscle." This hypothesis was communicated by Matteucci to the Academy of Sciences of Paris in 1847,* with an apologetic commentary concerning its vague and uncertain character. He appears to have been led to its conception partly in consequence of certain considerations which seemed to show that the phenomenon of "induced contraction" was owing to the discharge of electricity in the muscle in which the "induced contraction" was manifested—an idea originating with M. Becquerel—and partly in consequence of the analogy which he himself had found to exist between the law of contraction in muscle, and the law of the discharge in electrical fishes.

Observing that the muscles of frogs become more apt to contract under mechanical irritation when they are no longer under the control of the nervous centres, that the permanent contraction of rigor mortis is the state which supervenes when all signs of nervous influence are completely extinguished, and that cramps and other forms of excessive muscular contraction often happen spontaneously in paralyzed parts, Prof. Engel, in 1849, announced his views upon the influence of the nerves in the production of muscular motion in the following language:—"The function of the nerve is not to cause contraction of the muscle, but to a certain extent to counteract or oppose contraction. In the living organism, in which rest is an impossibility, a quiescent or inactive muscle or nerve is not to be imagined. The constant tendency of the muscle to contraction is prevented by the nerve, which continually endeavors to reduce the contraction of the muscle to an exact measure. From these two contradictory properties of the nerve and muscle results what is known as the condition of rest or equilibrium,—called also, in the muscle, tonicity. The removal of this equilibrium is followed either by motion or by paralysis. Motion is produced either by a reduction of nervous power, or by a direct increase in the contracting power of the muscles. Muscular paralysis results either from the immediate destruction of the contracting power of the muscle, or from an undue increase in the influence of the motor nerve over the muscle. The presence of a living nerve is indispensable to alternate muscular contractions, and these contractions continue so long as life is present in the nerves; when it disappears, the muscle contracts without opposition, giving rise to the condition known as rigor mortis."†

In 1852 Prof. Stannius, reflecting upon the manner in which *rigor*

---

* Comptes Rendus, March 17th, 1847.

† Ueber Muskelreizbarkeit. Zeitschrift der Kais-Kön, Gesellsch. des Aertze zu Wien, 1849.

*mortis* is relaxed by blood, announced in Vierordt's *Archiv*, "that the essential task of the motor nerves is to reduce the natural elastic volume of the muscular fibres, and to make their elasticity more complete; that active muscular contractions indicate a regulated, limited, and momentary remission of nervous influence; and that the customary reference to muscular irritability is entirely useless."*

It will thus be seen that a new and remarkable theory of muscular motion is beginning to assume a definite shape in the minds of physiologists.

Our author commences the discussion of this theory by laying down the following propositions :—

"1. That muscular contraction is *not* produced by the stimulation of any property of contractility belonging to muscle.

"2. That muscular elongation is produced by the simple physical action of certain agents, electricity and others, and that muscular contraction is the simple physical consequence of the cessation of this action.

"3. That the special muscular movements which are concerned in carrying on the circulation—the rhythm of the heart, and the movements of the vessels which are independent of the heart—are susceptible of a physical explanation when they are interpreted upon this view of muscular action."

These propositions are treated separately and at considerable length. In discussing the first, Dr. Radcliffe attempts to show that electricity, nervous influence, the blood, chemical and mechanical agents, light, heat, and cold, either singly or combined, do not produce muscular contraction, but on the contrary give rise to relaxation or elongation of the muscles.

To electro-physiologists it is well known that the researches of Galvani, Volta, Humboldt, and especially of Matteucci and Du Bois-Reymond, have demonstrated the existence of electric currents in muscles in a state of rest,—these currents proceeding sometimes from the origin toward the insertion of the muscle, and sometimes pursuing the reverse course. It is known, also, that the direction of the current may be changed by reversing the muscle, that the current is weakened by contraction of the muscle, and disappears altogether when the latter has lost its "irritability" and passed into the condition called rigor mortis. Having premised these facts, most of which he has experimentally confirmed, Dr. Radcliffe proceeds to set forth his peculiar views relative to the influence of electricity in muscular motion, in the following language, which we quote entire that our readers may have the whole argument before them :—

---

* Untersuchungen über Leistungsfähigkeit der Muskeln und Todtenstarre; Vierordt's Archiv für Physiol. Heilkunde; Stuttgart, 1852.

"On turning from these considerations to those which concern the action of the ordinary galvanic current upon muscle, it is to be expected that the existence of the muscular current is not to be ignored. And further, it is to be expected that certain reactions between the two currents will arise out of the particular course of the muscular current. It is no doubt true, as Dr. Du Bois-Reymond represents, that the muscular current passes from the end or ends to the sides of the muscular fibre; but it is also true that this current must complete its circle and return to the point from which it started. It is certainly true that the muscular current cannot have reached the ends of the fibre without having first reached the interior of the fibre, and that it cannot have reached the interior without having first passed through the exterior of the fibre. In other words, it is certainly true that the muscular current, in passing from the sides to the ends of the fibre, must have reached the interior by passing in a more or less *transverse direction across the exterior parts of the fibre.* Now, if such be the course of the muscular current, it is evident, first of all, that there is no way of passing the galvanic current through a muscular fibre in which this current will not cross the course of the muscular current. The galvanic current, it is true, will not always cross to the same extent, and to a certain point it may coincide with the course of the muscular current, as it will do when it passes along the interior of the fibre in the direction in which the muscular current is predominant—when it passes, for example, in an *upward* direction through the gastrocnemius; but if it coincides with the muscular current in its course along the *interior* of the fibre, it must cross the muscular current in that part of its course where, in order to reach the interior, it passes more or less transversely through the *exterior* of the fibre. And if the two currents cross in this manner, then must they clash, and (to an equivalent degree) neutralize each other. In a word, it is not enough to regard the muscle as a simple conductor, through which the galvanic current has to pass and take possession; but it is necessary to regard it as the seat of a current which must be overcome before the galvanic current can pass and gain possession. Nor it is not enough to say that the muscular current returns when the galvanic current is suspended. The muscular current does return at this time—of this there can be no doubt; but there is also another current which springs into existence at the same time, and this is the ordinary *reverse* current which arises out of the secondary polarity of some part of the circuit. Just then, as the muscle could not be looked upon as a simple conductor at the moment when the primary galvanic current began to pass, so now, it cannot be looked upon as a simple conductor at the moment when the muscular current returns to the muscle; for before the muscular current can regain possession of the muscle, it must contend with and overcome the reverse current above mentioned. It is evident, indeed, that there will be a clashing and equivalent neutralization between the muscular current and the reverse galvanic current at the moment when the circuit is opened, which is in every respect similar to that clashing and neutralization which took place between the muscular and primary galvanic current at the moment when the circuit is closed; and hence a moment in which electrical action is neutralized—a *moment of inaction,* as it may be called—may be supposed to precede both the establishment of the galvanic current and the return of the muscular current.

"What, then, it now remains to ask, are the phenomena which attend upon the passage of the galvanic current through the muscle, and how are they to be accounted for? The principal fact is, that a muscle contracts when the circuit is closed and when the circuit is opened, but not during the time that the current is passing through it. Thus, in an experiment of Professor Matteucci, in which a galvanic current was passed through the pectoral muscle of a pigeon from which every visible trace of nervous tissue had been carefully removed, the muscle was found to contract at the moment the circuit was closed and at the moment the circuit was opened, but not during the passage of the current; and this order was found to be altogether irrespective of the direction of the current. In this experiment it was also found that the contraction on opening the circuit was less marked than the contraction on closing the circuit; that both contractions became progressively less and less marked until they ceased altogether; and that one or both of the contractions might be revived for a short time after their extinction by increasing the strength of the current.

"Now in seeking for the explanation of these phenomena it is difficult, if not impossible, to find any reason for supposing that the contractions are due to any direct action of the current, natural or artificial. For what is the case? The case is that the muscle does *not* contract so long as the galvanic current continues to pass through it. The case is that the muscle does *not* contract so long as it is left to the undisturbed possession of the muscular current. But, on the other hand, there is no difficulty in connecting the contractions with that clashing and mutual neutralization of the muscular and artificial current, direct or reverse, of which mention has been made as attending upon the commencement and cessation of the passage of the artificial current through the muscle; for if contraction is absent when the artificial or natural current is manifestly present, and if contraction is present when the artificial or natural current is manifestly absent, then there appears to be only one course open, and that is to connect the contraction with the *absence* of the current.

"Upon this view, also, it is intelligible that the contractions should become less and less marked as the experiment proceeds, and that the contraction on opening the circuit should be less marked than the contraction on closing the circuit. Removed from the body, the muscle is found to yield fainter and still fainter indications of a current until these indications cease altogether, and hence it follows that the neutralizing reaction between the muscular current and the artificial current will fail more and more as the experiment proceeds,—will fail, because to diminish this current is evidently to diminish the amount of reciprocal neutralization,—and, so failing, that the contractions will become progressively less and less marked; for if the contraction is connected with the neutralization of electrical action in the muscle, the degree of the contraction must be directly proportionate to the degree of this neutralization. It is, also, intelligible that the contraction on opening the circuit should be less marked than the contraction on closing the circuit, for the lapse of time and the disturbing influence of the artificial current upon the electromotive elements of the muscle will combine to weaken the muscular current—will combine, that is, to render the contraction on opening the circuit less marked than the contraction on closing the circuit; for—to repeat what has just been said—to weaken this current is to diminish those neutralizing reactions between the two currents, which, upon this view, are directly measured by the degree of the contraction.

"Nay, it is in some degree possible, upon the same view, to explain the fact that the contractions should be reproducible, after they have once ceased, by increasing the strength of the artificial current; for in order to explain this difficulty, it is only necessary to suppose—and the supposition is certainly allowable—that the weaker current had only penetrated the muscle superficially, and that the stronger current had penetrated more deeply and reacted with the current of muscular fibres which had been beyond the range of the weaker artificial current. This supposition is certainly allowable, for current electricity, although of very low tension, must yet possess some tension, and on that account it must agree in some degree with electricity of tension in preferring to occupy the surface of the body which serves as a conductor.

"There is, no doubt, much that is not altogether satisfactory in some of these speculations, and it cannot well be otherwise until a clearer knowledge of electricity is brought to bear upon the subject; but at the same time it must be evident that there is no reason for believing that muscle is stimulated into the state of contraction by electricity, and that this state of contraction passes off when this stimulation is at an end; and it may also be allowed that there is some reason for concluding that muscular elongation, and not muscular contraction, is the state which is produced by the action of electricity upon the muscle.

"Nor is there any objection to this view in the physical condition of the contracted and uncontracted muscle. At first sight, indeed, it would appear as if a stimulus had ceased to act when the muscle passes out of the contracted state, and it is no easy matter to get rid of this idea; but Professor Ed. Weber has shown, not only that the hardness of muscle in a state of contraction is simply owing to the straining of the muscle upon its attachments, but also that the contracted muscle is even softer than the uncontracted muscle when it is cut loose from these attachments. In other words, he has shown that the condition of the uncontracted muscle may be more correctly represented by the term *elongation* than by the term *relaxation*,—and hence the mere physical condition of the muscle does not oppose any objection to the idea that muscular elongation, and not muscular contraction, is produced by the action of electricity upon muscle."

Our author next endeavors to show that muscular contraction is not produced by the stimulation of nervous influence. He tells us that the term "nervous influence" is a convenient one, which includes certain agents thought to be vital, and others which fall within the scope of physical inquiry. Believing that there is one common force underlying all these agents, whether physical or vital, and that of them all electricity is the one most easily investigated, he proceeds to interpret the mode in which muscle is affected by "nervous influence" from an electrical point of view.

To Dr. Du Bois-Reymond belongs the honor of demonstrating for the first time in a masterly and complete manner, the existence of actual electrical currents in nerves. By means of the small galvanometer employed by him in the investigation of muscular currents, he demonstrated the existence of a current passing from the end to the side of the nerve. Reversing the position of the nerve does not affect the direction of the current, but when the nerve is doubled into a loop, and the ends brought together, then two currents are observed to set from the ends of the nerve. The phenomena of the "nerve current" appear to be precisely similar to those of the "muscular current"—except that no evidence of a current could be found between dissimilar points in the transverse section of the nerve. This difference our author is inclined to attribute to the smallness of the nerve. These electrical phenomena may be observed in purely motor and sensory nerves, as well as in mixed nerves like the ischiatic. The nerve current, like the muscular current, dies out *pari passu* with the irritability, and the extinction is complete when the latter has entirely disappeared. The extinction of the current does not appear to be preceded by any reversal of its direction. According to Dr. Radcliffe, there is such a reversal if the nerve current be examined in a nerve which retains its connection with the muscle, and this equally whether the irritability has been allowed to die out gradually or whether it has been suddenly exhausted by a series of alternating shocks from an induction coil. He assures us that this reversal is in some way connected with the reversal of the muscular current, for it ceases when the nerve is removed from the muscle, and is never manifested when the nerve current is investigated from the beginning in a separated nerve. From the observations of Dr. Du Bois-Reymond it also appears that the nerve current becomes enfeebled during muscular contraction. In this respect it resembles the muscular current. When a portion of a nerve is included in the circuit of a simple galvanic apparatus a current is set up not only in this portion, but also in every part of the nerve. At the same time the muscles of the part are seen to contract when the circle is opened and closed, and to remain elongated so long as the current continues to pass.

"At first, no very marked differences in the degree of these two contractions are produced by changing the direction of the current. Afterwards, when the excitability of the nerve has become diminished, there begin to be certain differences in the degree of these contractions, which differences are determined by the direction of the current in the nerve,—the law being, first, that the contraction on opening the circuit is more marked than the contraction on closing the circuit, *when the current is said to be 'inverse'*—when, that is, it passes from the expansion to the origin of the nerve; and second, that the contraction on closing the circuit is more marked than the contraction on opening the circuit,

*when the current is said to be 'direct'*—when, that is, it passes from the origin to the expansion of the nerve.

"If the current be *direct*, that is, from the origin of the nerve to its expansion, and if it be passed for some time continuously, and the circuit then opened and closed, the opening and closure of the circuit are not attended by any contractions in the muscular fibres; but if the current be *inverse*, that is, from the expansion to the origin of the nerve, this is not the case; and provided the galvanic current be not too powerful, the current may be passed through the nerve for a much longer time than in the last instance, and the contractions may still attend upon the opening and closure of the circuit. Instead of exhausting the excitability of the nerve, as did the *direct* current, it would indeed seem as if the *inverse* current had augmented this excitability; and this was the opinion of Professor Matteucci, who first directed attention to these differences in the effects of the direct and inverse currents. At any rate, there is a *marked* contraction on opening the circuit after the *inverse* current has been passing for some time through the nerve.

"At first sight, perhaps, it is difficult to understand how it is that the artificial current should act so differently according as it is passed up the nerve or down the nerve, but on referring to the particular reactions which must take place between the galvanic current and the nerve current under these circumstances, this difficulty is done away with.

"*When the circuit is closed*, the galvanic current will pass across the course of the nerve current, and so passing it will neutralize and receive an equivalent amount of neutralization from the nerve current. And thus an instant of neutralization, a moment of inaction, will precede the establishment of the galvanic current in the nerve. *When the circuit is opened*, the galvanic current will be suspended, and the nerve current will return to the nerve; but before the nerve current can thus return, it will have clashed for an instant with that *reverse* current which springs into momentary existence and traverses the nerve and other portions of the galvanic circuit upon the cessation of the primary galvanic current. In other words, the re-establishment of the nerve current after the cessation of the artificial current, will also be preceded by an interval of inaction, in which the first installments of the nerve current and the momentary reverse current suffer that reciprocal neutralization which arises out of the differences of their direction. And hence it would appear that there is some neutralization of electrical action in the nerve at the moment of closing and at the moment of opening the circuit, and that the nerve is only under full electrical action either when the artificial current is passing through it, or when the nerve is left to the influence of its own current.

"Looking at muscular action, then, in relation to the *electrical* changes of the nerves concerned in this action, it would seem that muscular elongation is coincident with the *action* of natural or artificial electricity upon the nerve, and that muscular contraction is connected in some way with the diminution or annihilation of this action; and no reason has yet arisen which can support the idea that any vital property of nerve has been called into active exercise during the time of contraction by the 'stimulus' of electricity."

Dr. Radcliffe next adduces from the writings of Brown-Séquard, Martin Magron, and Engel, a number of facts and experiments to show that muscular contraction is not produced by any stimulation derived from the nervous centres. He thinks that the will influences muscular action not so much by imparting anything to the muscles, but by withholding something from them.

Dr. Radcliffe next proceeds to show that muscular contraction is not produced by the stimulation of blood. For the proofs of this proposition he appeals first to comparative anatomy, which teaches us that the degree and duration of muscular contraction are greater in the voluntary muscles of fishes and reptiles than in the voluntary muscles of mammals and birds; greater in involuntary than in voluntary muscles; and greater in animals in a state of hybernation than in a state of activity. He refers next to the experiments of Brown-Séquard* and Stannius† in proof of the fact that rigor mortis may be relaxed, and the lost irritability restored to the muscle by the injection of blood into the vessels. The views of Dr. Brown-Séquard, concerning the different effects produced by arterial and venous blood upon muscular tissue, are then animadverted upon. This observer has been led by many experiments of his own to the conclusion that arterial blood in providing for the nutrition of the muscle also supplies it with contractile power, with motility or irritability; while the venous fluid, in virtue of its carbonic acid acts as a stimulus, and serves to arouse this irritability and bring it into action. Some of the arguments and experiments upon which this opinion is based are briefly referred to by our author, who thinks that "they are very far from showing that muscle is stimulated to contract by venous blood." He questions whether the convulsions of asphyxia are not rather due to the want of the stimulus of red blood than to any stimulus derived from the black blood with which the system has become charged; and he observes, what is certainly true, that the muscles are similarly convulsed when an animal is bled to death. The more powerful contraction of the left ventricle during the first moments of asphyxia—a fact which Brown-Séquard cites in favor of his theory, and which he thinks is due to an increase in the stimulating influence of venous blood—is attributed by Dr. Radcliffe to "some impediment to the free flow of blood through the capillaries of the systemic circulation—an impediment by which the systole of the ventricle is made to expand itself with greater force upon the coats of the intermediate arteries." Dr. Radcliffe also reminds us "that the uterus has often contracted and expelled its contents when a pregnant animal has been bleed-

* Comptes Rendus, 9 et 25 Juin, 1851.

† Archiv für Physiologische Heilkunde; Stuttgart, 1852. Heft. i.

ing to death. And there are several cases on record in which the human uterus even has expelled its burden after the mother has yielded to the utter syncope of actual death."

In the next section, our author attempts to show that muscular contraction is not produced by the stimulation of any mechanical agent. His arguments upon this point are so purely speculative and hypothetical, and withal so imperfect and unsatisfactory even to himself, as he acknowledges, that we are constrained to pass them over without further notice.

He thus briefly alludes to the influence of light upon muscular contraction:—

"Regarding the question generally, contraction would seem to be favored by darkness rather than by light. It is in the darkness, and not in the light, that contraction takes place in the irritable cushions of the untouched sensitive plant; and it does not seem to be far otherwise with the iris, for it is as easy to suppose that the radiating fibres of this organ elongate under the influence of light, and in that way close the pupil, as to suppose that this curtain is closed by sphincter fibres, which have a very doubtful existence. At all events, this explanation is supported by the authority of Bichat; it equally accounts for the phenomena: and it harmonizes with the known influences of light upon the cushions of the sensitive plant."

Muscular fibre may be subjected to considerable variations of temperature without contracting; but if its temperature be elevated or lowered very much, it immediately contracts.

"Now," says Dr. Radcliffe, "there is reason to believe that the 'muscular current' is more or less suspended by a temperature which is higher or lower than a given point, and not by intermediate degrees of temperature, and that the presence or absence of contraction in these experiments may be accounted for by the suspension or non-suspension of the 'muscular current.' Professor Matteucci has shown that the 'muscular current' is suspended by a low temperature, and hence, according to the premises, it is intelligible that the muscle may contract under a sufficient degree of cold. On the other hand, I find that the muscular current is weakened to the last degree, or altogether extinguished by the amount of heat which is sufficient to produce contraction in the muscle. In this experiment I placed the gastrocnemius of a frog across the cushions of the galvanometer, and having laid the end of a small straight band of watch-spring upon it, I raised the temperature of the band by applying a spirit-lamp to the other end. It is intelligible, therefore, that muscle should contract under a temperature which is higher or lower than a given point, and that it should not contract under those intermediate degrees of heat which are not sufficient to weaken the 'muscular current' to the point which allows of contraction."

In the seventh section, after attempting to show that muscular contraction is not produced by the stimulation of any chemical or analogous agency, our author thus sums up his argument:—

"Reviewing the whole evidence, then, there appears to be no reason why *rigor mortis* may not be taken as the type of muscular contraction in general. For what is the case with respect to this form of muscular contraction? The case is simply this. As long as there is any trace of that action of which the 'muscular current' is a sign, so long is there no *rigor mortis.* As long as there is any trace of that action of which the 'nerve current' is a sign, so long is there no *rigor mortis.* If this action dies out speedily, as in persons in whom the vitality of the frame has been exhausted by long life, or by chronic disease, such as consumption, the muscles become speedily rigid; if this action dies out slowly, as in persons who have been cut down suddenly in the full glow of life, the muscles are equally slow in becoming rigid. Once contracted, moreover, the muscles remain contracted until they break up in the ruin of final decay,—an event which happens most speedily in the case where the muscle retains its physical integrity least perfectly. And this is precisely as it should be according to the premises, for according to the premises all that is necessary to the commencement of *rigor mortis* is the cessation of that action of which electricity is a sign, and all that is necessary to its continuance is the absence of this action and the physical integrity of the muscular fibre. In a word, it is possible to explain those unexplained and seemingly contradictory facts which constitute the distinctive features of that contraction into which the muscles pass after death; and hence *rigor mortis* may be accepted, not only as a type of muscular contraction in general, but as an *experimentum crucis* in favor of the proposition—that *muscular contraction is not produced by the stimulation of any contractile power belonging to muscle.*"

Thus much for the first proposition. In attempting to establish the second—that muscular elongation and contraction are simple physical phenomena—a comparison is instituted by our author between the changes which a muscle undergoes in the act of contracting, and those purely physical alterations in form and volume exhibited by many crystalline bodies when subjected to the influence of heat. Under this influence these crystals increase in length and lose in breadth, with but slight change in volume. As the muscle does not alter its volume, but gains in breadth what it loses in length, the analogy sought is imperfect and partial. But Dr. Radcliffe refers us, for a more direct and satisfactory illustration of muscular contraction, to the changes produced in a bar of iron under the influence of magnetism,—changes which consist, as in the case of a contracting muscle, in an increase of length and loss of breadth without any alteration of volume. For the knowledge of these interesting facts concerning magnetized iron, we are chiefly indebted to Mr. Joule, of Manchester, for a detailed account of whose very numerous and exact experiments the reader is referred to the *Philosophical Magazine* for February and April, 1857.

"The changes which take place in a bar of iron under the influence of magnetism are paralleled, therefore, by the changes which take place in muscular fibre, for while the fibre increases in length and loses in breadth, under the influence of electricity and its associate agencies, and loses in length and gains in breadth when this influence is withdrawn, so likewise does the bar of iron increase in length and lose in breadth under the influence of electricity, and lose in length and gain in breadth when this influence is withdrawn. And not only is this parallelism preserved in the changes of shape and in the agencies concerned, but it is preserved in that point which is so characteristic of muscle, namely, the *suddenness* with which the contracted and elongated states may alternate upon each other,—for in Mr. Joule's experiments the bar was seen and heard and felt to *jump* into the longer form, and then to jump back again into the shorter form, as the electricity was communicated to or withdrawn from the coil."

It would appear that a contracting muscle and a magnetized bar of iron exhibit analogous, if not indeed homologous phenomena, as Dr. Radcliffe contends. But several interesting questions here present themselves. How, for example, can we explain the diminished degree of shortening exhibited in the post-mortem contraction of a muscle, except upon the supposition that the contractile power is a vital endowment? How may we account for the loss of contractile power consequent upon death? Why does the muscle lose its power in proportion to the degree of contraction? If contraction is a mere physical process, and not a vital act, why does the muscle undergo disintegration and waste in a degree proportionate to the number and activity of its contractions? Dr. Radcliffe anticipates and attempts to clear away these objections to his theory by various arguments, which, though more or less plausible, are by no means of sufficient weight to carry conviction to our mind.

The third and last proposition which our author attempts to establish is, as we have already seen, "that the special muscular movements which are concerned in carrying on the circulation are susceptible of a physical explanation."

In the discussion of this proposition an attempt is first made to solve the "riddle of the heart's action"—that mysterious riddle which from the days of Harvey down to the present time has so profoundly taxed the ingenuity, experimental skill, and reasoning powers of so many, and such able physiologists.

Dr. Radcliffe contends that the systole of the ventricles is not produced by the stimulation of arterial blood, but that, on the contrary, the blood induces the diastole while it antagonizes the systole. He thinks that—

"The oxygen of the arterial blood, injected into the coronary arteries by the systole of the left ventricle, and acting upon the muscular and nervous elements of the coats of the heart, produces the diastole by rousing (among other things) the muscular and nerve currents, or, in other words, by rousing the polar condition of the muscular and nervous fibres; and that the systole follows the diastole in consequence of the failure of these currents, when the arterial blood has parted with its oxygen, and so ceased to be sufficiently stimulating. And if, under ordinary circumstances, the blood acts in this manner, then there appears to be no great difficulty in understanding how it is that the heart, or a portion of the heart, may go on pulsating after removal from the body. For why should oxygen dissolved in the blood act differently from oxygen diffused in the air? Why, for instance, should not the air, which bathes the surface and permeates every interstice, provoke a diastolic state in the separate heart or a fragment of the same, by rousing that polar condition which in the nerves and muscles is designated under the name of the nerve and muscular currents? Why may not the systolic state supervene upon this diastolic state when the polar condition fails, in consequence, as it were, of the arterial air having become converted into venous air? And why, again, should not the diastole return after every systole, so long, that is, as the muscle is capable of responding to the action of the oxygen, for it may well be supposed that the commotion of the systole will displace the venous air and bring the muscular and nervous tissues into relation with fresh quantities of arterial air? Assuredly there is no evident reason to the contrary, and there is one reason why this view should be received, and this is to be found in the fact that the rhythm is rendered more rapid, and even revived for some time after its actual cessation, by placing the heart in oxygen instead of atmospheric air, and that it is brought to a stop by placing the heart in a vacuum or by immersing it in hydrogen or nitrogen or carbonic acid.

"It may be assumed, also, that the same view is no less applicable to the explanation of other kinds of rhythmic motion, for it is easy to perceive that these different forms may depend, on the one hand, on differences of structure—differences in the kind of irritable fibre or substance, in the presence or absence of nerves, in the number of blood-vessels, or in the amount of exposed surface—and, on the other hand, upon the different rates and degrees of oxidization which are consequent upon these differences of structure."

Finally, he maintains that the muscular coats of the blood-vessels are stimulated to contraction neither by blood, nor nervous influence, nor heat, but that these agents really antagonize contraction and produce elongation of the vascular muscular fibres, and consequent expansion of the vessels.

The second part of the work before us is divided into five chapters, which are severally devoted to the consideration of Simple Epilepsy, Tremor, Simple Convulsion, Epileptiform Convulsion, and Spasm.

In the first chapter, after discussing the history and symptoms of simple epilepsy, Dr. Radcliffe attempts to show that the pathology of this affection is in keeping with the theory of muscular contraction above set forth. He assures us that post-mortem examinations reveal no constant anatomical lesions in simple epilepsy; that the cerebral congestion sometimes discovered is rather consequent upon than a precursor to the disease; and that in cases of the latter, complicated with insanity, the signs of inflammation of the brain and its membranes are clearly referable to the mental disorder, and "this for no other reason than they are as common, or more common in insanity without epilepsy, than in insanity with epilepsy." He thinks, however, that certain other anatomical characters which belong to dementia, such as pallor of the gray matter, softening, induration, atrophy, dropsical effusion, etc., furnish some grounds for supposing that epilepsy is caused by them, since the demented state is intimately connected with convulsive disorder, for if a demented person be not epileptic, he is almost sure to be afflicted with palsied shakings, or cramps, or spasms, in one form or another. He maintains that—

"The phenomena connected with the vascular system are altogether opposed to the idea of arterial excitement in epilepsy. It would seem, indeed, as if the spasms, as well as the loss of consciousness and sensibility, were connected with a deficiency of arterial blood; for, in the first place, there is a state closely approaching to syncope, and in the next place, a state of positive suffocation, or arrested arterialization of the blood. It is not improbable, also, that the blood may have been previously rendered less stimulating than it ought to be by the imperfect exercise of some organ of excretion. The phenomena, indeed, are in harmony with the previous considerations respecting muscular action, for, according to them, the office of the blood is to antagonize contraction, not to cause it.

"Nor does there appear to be any reason for supposing that venous congestion has a more important part to play in the production of epilepsy than that which has been assigned to arterial injection. No doubt the veins of the brain and head generally are congested from a very early moment, but there is a moment antecedent to this in which the death-like paleness of the face—in many cases at least—is a sufficient proof that the veins were emptier than usual before they became congested. Indeed, it may be supposed that this was the case in the majority of instances, if not in all, for there would seem to be no way of accounting for the instantaneous loss of consciousness and sensibility (which is in reality the first phenomenon of the fit) except upon the supposition of some sudden failure in the supply of blood to the great nervous centres."

To the inefficient action of these centres is ascribed the morbid irritability of the nervous system so well marked in the epileptic.

In the treatment of simple epilepsy, Dr. Radcliffe strongly advocates a nutritious animal diet, a liberal allowance of alcoholic stimulants, the free use of coffee, continence in sexual matters, the avoidance of much mental exertion, whether in study or otherwise, and the employment of but little bodily exercise. The first and last of these particulars are opposed to the well-known views of Dr. Brown-Séquard concerning the causes and treatment of epilepsy. This observer ascertained experimentally that in epileptic animals the number of fits in a given time is generally in direct proportion to the quantity of food taken; and that there is an inverse proportion between the amount of exercise and number of fits.

The remedies most commonly employed at present in the medical treatment of epilepsy are oxide of zinc, ammonio-sulphate of copper, and nitrate of silver. In the remedial efficacy of these substances our author has no faith whatever. He has used them without success. His friend, Dr. Marcet, has given the oxide of zinc an extended trial at the Westminster Hospital, but with similar results. In the Bicêtre, M. Moreau treated eleven adult epileptics with the oxide, but failed to cure any of them. M. Herpin, who originally introduced the zinc and copper treatment, has himself become much less confident of the efficacy of the oxide of zinc than he was in 1852.

"Now these three remedies, zinc, copper, silver, belong to the group of diamagnetic bodies—of bodies, that is to say, which tend to arrange themselves at right angles across the magnetic meridian. They belong, indeed, to a group in which some of the companion metals are bismuth, antimony, zinc, tin, sodium, mercury, lead, silver, copper, gold, arsenic. They belong to a group of metals, that is to say, which are all more or less sedative in their action, and this possibly may be one explanation why the result of their use in epilepsy has not been more satisfactory; for after what has been said upon the pathology of this affection, it is scarcely to be expected that a sedative remedy of any kind will be that which best suits the requirements of the case. And if this inference be correct, is it to be expected that a remedy may be found among the magnetic bodies—among bodies of which iron, nickel, manganese, cobalt, take the highest rank?"

Iron has been recommended by Dr. Watson, and quinine by Rostan and Piorry. Dr. Radcliffe has used both these articles with great success. Latham, Foville, Perceval, and Watson, speak very highly of the use of oil of turpentine in epilepsy, in doses varying from half a drachm to a drachm every six hours. Dr. Radcliffe tells us that he has had abundant evidence of its value in this disease. He attributes its efficacy to its stimulating effects upon the circulation. The nauseous taste of this article, however, and the irritation which it occasionally sets up in the urinary and generative organs, constitute a serious objection to its use. Valerian, originally recommended by Aretæus and Dioscorides, and used ever since, is considered by our author as decidedly inferior in value to the turpentine. He speaks disparagingly also of strychnia, belladonna, digitalis, indigo, cotyledon umbilicus, selinum palustre, poudre de Neufchâtel, (*taupe grillée* or fried mole,) and bromide of potassium. All these substances have been exhibited in epilepsy, but with occasional benefit only. Compression of the carotids, advocated by Romberg and Sieveking; tracheotomy, proposed and practiced by Marshall Hall; and cauterization of the larynx, suggested and performed by Drs. Brown-Séquard and

Ebenezer Watson, meet with but little approval from our author. At times, and in certain peculiar cases, they may be of use, but they never can be relied upon as valuable methods of relief. Dr. Radcliffe strongly recommends naphtha, camphor, chloric ether, Hoffman's anodyne, and aromatic spirits of ammonia, either singly or combined with each other, or in combination with iron and quinine, according to the special indications of the case. Under the use of these remedies he found that the general health was improved in every case, the spirits became more equable and cheerful; headache disappeared; the pulse acquired increased power, and the warmth of the body was maintained with less difficulty and greater uniformity. An improvement in the fit was always contemporaneous with this improvement in the general health, at least in this—that the coma attending the fit became less and less profound, and the subsequent oppression or bewilderment or headache less and less distressing. He assures us also that in many cases the fits were not only lessened in severity by being deprived of their most ominous characteristic, coma, but the intervals between the fits were lengthened to such a degree as to afford good ground for supposing that the fatal habit might be altogether broken by a continuance of the same treatment.

